# Supplementary material for: Microsatellite Instability Testing and Prognostic Implications in Colorectal Cancer
Source: Cancers (Basel). 2024 May 25;16(11):2005. doi: 10.3390/cancers16112005 (PMC11171323; doi:10.3390/cancers16112005)
Supplement: Supplementary file 1 [file cancers-16-02005-s001.zip › cancers-2988159-supplementary.pdf]

Table S1. The distribution of dMMR expression in CRC patients.

dMMR Type	n	Percentage
MLH1, PMS2	10	45.5%
MSH2, MSH6	3	13.6%
PMS2	3	13.6%
MLH1	5	22.7%
MSH6	1	4.6%
Total	22	100.0%

Abbreviations: dMMR, MMR deficiency
